# Blood Flow Restriction in Athletic Populations—Part 2: Applications in Resistance Training Across the Loading Spectrum

**DOI:** 10.3390/jfmk11020176

**Published:** 2026-04-27

**Authors:** Chris Gaviglio, Christian J. Cook, Stephen P. Bird

**Affiliations:** 1 School of Health, Psychological and Medical Sciences, University of Southern Queensland, Ipswich 4305, Australia; 2 Institute for Health, University of Southern Queensland, Ipswich 4305, Australia; 3 Biomedical Discipline, School of Science and Technology, University of New England, Armidale 2350, Australia

**Keywords:** blood flow restriction, resistance training, hypertrophy, strength, power development, velocity-based training, athletic performance

## Abstract

**Background:** Blood flow restriction (BFR) resistance exercise has emerged as a training methodology capable of inducing muscular adaptations comparable to traditional high-load training despite substantially lower mechanical loads. While low-load BFR protocols (20–50% 1RM) are well-established, emerging evidence supports applications across the full loading spectrum, including moderate-to-high loads (>50–90% 1RM), contralateral training effects, and proximal–distal adaptations. In this second installment of the Blood Flow Restriction in Athletic Populations series, we review current evidence on BFR resistance exercise in athletic populations, with emphasis on morphological, neuromuscular, and functional adaptations across diverse application contexts. **Methods:** A narrative review of research examining BFR resistance exercise in trained and athletic populations was conducted via a PubMed/MEDLINE search. Search terms: (“blood flow restriction” OR “BFR” OR “occlusion training” OR “KAATSU”) AND (“resistance training” OR “resistance exercise” OR “strength training”) AND (“athletes” OR “athletic” OR “trained” OR “elite” OR “sport”) AND (“cross-education” OR “contralateral” OR “cross transfer” OR “proximal” OR “distal”). Studies investigating low-load (20–50% 1RM) and moderate-to-high load (>50% 1RM) protocols, contralateral cross-education effects, and proximal–distal adaptations were evaluated. Primary outcomes included muscle hypertrophy, strength, power, and sport-specific performance measures. **Results:** Low-load BFR resistance exercise has been shown to produce significant improvements in muscle hypertrophy and strength gains over 4–12 week interventions compared to low-load control conditions. Moderate-to-high load BFR enhanced barbell velocity and power output, particularly at loads > 80% 1RM with intermittent inflation protocols. Contralateral and cross-transfer effects of BFR training demonstrate variable efficacy across muscle groups, with the most consistent evidence supporting cross-transfer enhancement of training adaptations when BFR is applied to one body region while exercising another. Proximal BFR application induced adaptations in both proximal and distal musculature, suggesting systemic mechanisms beyond local vascular restriction. **Conclusions:** BFR resistance exercise represents a versatile training modality producing meaningful morphological and neuromuscular adaptations across the loading spectrum. Contralateral and proximal–distal effects expand practical applications for injury rehabilitation and targeted adaptation. These findings support BFR integration within periodized training programs when mechanical load management is prioritized.

## 1. Introduction

In Part 1 of this series, we established the physiological basis of blood flow restriction (BFR) training alongside key safety considerations, including contraindication screening, risk stratification, and safety application principles. We also outlined the methodological frameworks for BFR use, including individualized pressure determination, cuff selection, and real-time monitoring strategies to optimize safety and effectiveness. In this second installment of the Blood Flow Restriction in Athletics Populations series, we review BFR within resistance exercise across the full loading spectrum. Readers unfamiliar with general BFR methodology are encouraged to consult Part 1. In this section, the evidence for low-load and moderate-to-high-load BFR resistance exercise is examined, with an emphasis on protocol design, performance outcomes, and applied programming considerations relevant to coaches and practitioners.

Resistance exercise combined with blood flow restriction (BFR-RE) has been demonstrated to enhance both strength and hypertrophy across a wide spectrum of loading intensities. Early research focused on how low-load protocols (20–40% 1RM) influence muscular adaptations [1,2,3]. Recent studies demonstrated safe and efficacious application of BFR-RE during moderate-to-high loadings (>50% 1RM) [4,5,6,7], thereby expanding its utility in athletic populations and rehabilitation contexts [8]. The underlying mechanisms driving BFR-RE adaptations involve a synergistic interaction between mechanical tension, metabolic stress, and cellular swelling, with downstream effects on mTOR pathway activation, muscle protein synthesis, and satellite cell recruitment [9]. Notably, low-load BFR-RE can elicit muscle responses similar to high-intensity lifting, which suggests comparable neuromuscular engagement despite reduced external load [10].

**Figure 1:**
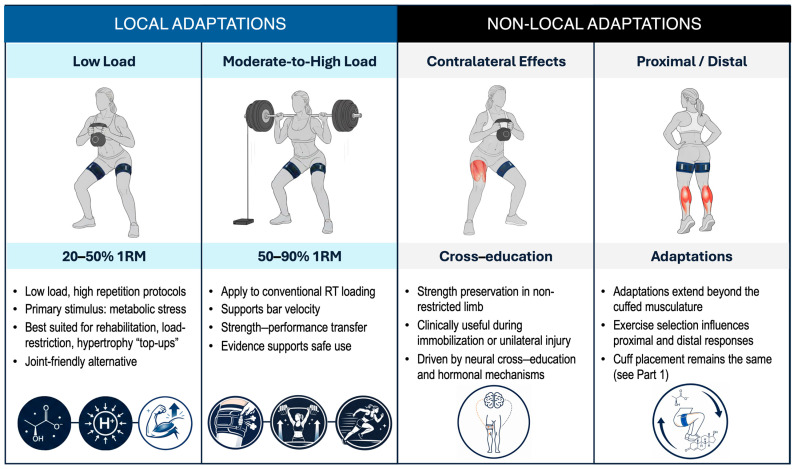
Local and non-local adaptations to BFR exercise across loading conditions. Schematic representation showing direct muscular adaptations in restricted limbs during low-load (20–50% 1RM) and moderate-to-high load (>50% 1RM) BFR protocols; contralateral cross-education effects showing ipsilateral strength preservation during unilateral BFR training; and proximal–distal adaptations demonstrating systemic effects beyond the site of cuff restriction. Arrows indicate direction and magnitude of adaptive responses. RT, resistance training; 1RM, one-repetition maximum.

To contextualize the acute response and chronic adaptations discussed in this paper, BFR-RE can be broadly defined according to both the loading intensity and spatial distribution of training adaptations. Across the resistance exercise loading continuum [11], BFR may be applied during low-load resistance exercise, or during moderate-to-high load resistance exercise, using intermittent cuff application to support strength- and performance-related outcomes. In addition to local adaptations within the restricted limb, BFR-RE has also been shown to elicit non-local responses, including contralateral cross-education effects and proximal–distal adaptations extending beyond the cuff site. These local and non-local adaptations, presented in Figure 1, provide a framework for the subsequent sections of this review. This framework provides the structural basis for the present review, with subsequent sections synthesizing evidence according to loading condition and the spatial distribution of adaptations.

## 2. Methods

### 2.1. Search Strategy

A narrative review of research examining BFR resistance exercise in trained and athletic populations was conducted. A comprehensive literature search of PubMed/MEDLINE was performed using combinations of the following search terms: (“blood flow restriction” OR “BFR” OR “occlusion training” OR “KAATSU”) AND (“resistance training” OR “resistance exercise” OR “strength training”) AND (“athletes” OR “athletic” OR “trained” OR “elite” OR “sport”).

To capture literature examining non-local adaptations, additional searches included terms such as (“cross-education” OR “contralateral” OR “remote adaptation” OR “proximal” OR “distal”). The reference lists of the retrieved articles were also manually searched to identify additional relevant studies. As this review was designed as a narrative synthesis focused on applied resistance training, studies were selected based on their relevance to BFR-RE protocol design, adaptations, and practical application.

### 2.2. Study Selection

Studies were included if they met the following criteria:

- Low-load BFR-RE protocols (20–50% 1RM).
- Moderate-to-high load BFR-RE protocols (>50% 1RM).
- Study design: Randomized controlled trials, controlled trials, case studies, or within-subjects designs.
- Contralateral cross-education effects.
- Proximal–distal adaptations.
- Studies involving trained or athletic populations (individuals with ≥1 year of structured resistance training or competitive sport participation).

Exclusion criteria:

- Studies exclusively in untrained, clinical, or elderly populations.
- Studies without a clear BFR methodology.
- Studies not available in English.

While the primary focus of this review was research conducted in trained or athletic populations, additional studies examining non-local adaptations to BFR resistance exercise (e.g., contralateral, proximal, or distal effects) were considered where relevant mechanistic insights or training implications could be inferred for athletic populations.

A single reviewer (primary author) conducted the search and study selection, with consultation among all authors for ambiguous cases. We acknowledge that this selective approach may introduce bias toward positive findings and well-established research groups and may favor studies with positive or applied outcomes. 

### 2.3. Data Extraction and Synthesis

For each included study, we extracted:

- Population characteristics (training status, sport, sample size);
- BFR-RE protocol details (cuff pressure, load, volume, frequency, duration);
- Outcome measures (hypertrophy, strength, power, performance);

Where reported in the original publications, effect sizes were extracted to assist in the interpretation of the magnitude of responses. Standardized effect sizes were not calculated independently due to inconsistent reporting across studies and the limited availability of original data. As this review was designed as a narrative synthesis, no formal quality assessment tool or pooled statistical analysis was applied. Findings were synthesized narratively and organized thematically by loading condition and adaptation type.

### 2.4. Coverage of Available Literature

While this review is narrative in design, our search strategy was comprehensive for the specific population of interest (trained individuals and competitive athletes). A scoping assessment of the literature revealed that studies of BFR-RE in athletic populations (as opposed to untrained or recreationally active individuals) are relatively limited in number compared to the broader BFR literature. Our search strategy, combined with manual reference list screening, captured the majority of studies meeting our inclusion criteria. Therefore, we provide practitioners with a comprehensive overview of the current evidence base in trained and athletic populations, organized by loading condition and adaptation type for practical application. While we did not employ formal systematic review methodology (PRISMA guidelines, multiple independent reviewers, quality assessment tools), the relatively focused nature of the literature in athletic populations meant that our narrative approach yielded thorough coverage of available evidence.

## 3. Low-Load BFR Resistance Exercise (20–50% 1RM)

Low-load BFR (LL-BFR) resistance exercise (20–50% 1RM) represents the most established and widely researched form of BFR training [12,13,14]. It has consistently been shown to produce muscle hypertrophy and strength improvements comparable to traditional high-load resistance exercise, despite substantially reduced mechanical stress. For athletes managing high training volumes, recovering from injury, or during in-season phases where heavy loading may compromise performance or recovery, this approach offers a viable and effective alternative. The evidence base spans diverse athletic populations, including team sport athletes (rugby, football, netball), individual sport athletes (track and field, weightlifting), and resistance-trained individuals, demonstrating broad applicability across training contexts (Table 1).

Foundational studies have demonstrated that low-load BFR-RE can induce muscle hypertrophy and strength gains comparable to traditional heavy resistance training. For example, Takarada et al. [1] observed significant increases in bilateral knee extension torque (14.3%) and muscle cross-sectional area (12.3%) following 8 weeks of BFR-RE at 50% 1RM in elite rugby players, while Abe et al. [2] reported improvements in 1RM leg press (9.6%) and sprint performance in trained track and field athletes performing twice-daily sessions at 20% 1RM over just 8 days. Five weeks of low-load BFR-RE at 20% 1RM improved isometric strength (9.3–13.3%), endurance (87.7%), and muscle thigh cross-sectional area (6.6%) in female netball players [3]. Collectively, these studies support low-load BFR as a potent stimulus to promote hypertrophic adaptations, strength expression, and sport-specific performance while minimizing mechanical joint stress. Across these studies, hypertrophic responses (typically ~4–12% increases in muscle cross-sectional area) and strength gains (~6–15% improvements in 1RM) appear to be achieved using loads of 20–50% 1RM, relatively high repetition volumes, and short inter-set rest periods. Higher training frequencies and protocols incorporating sets to or near failure may further augment these adaptations.

Primary considerations around BFR-RE protocols include cuff inflation pressure (typically 50–80% AOP), total training volume (sets and repetitions), inter-set rest, and cuff inflation modality (i.e., continuous vs. intermittent). For low-load BFR-RE, the goal is to potentiate physiological responses rather than replicate the mechanical outcomes associated with traditional high-load strength training. Although no universal protocol exists for scaling BFR-RE parameters with incremental loads, it is plausible that optimal results arise from the cumulative effect of both mechanical stress (external resistance) and metabolic stress (occlusion-induced hypoxia). Accordingly, at low loads (20–40% 1RM), protocols should favor high repetitions, continuous pressure, and higher relative restriction pressures (≈70–80% AOP) to maximize the metabolic environment for muscle adaptation. As training loads increase (>50% 1RM), the emphasis may shift toward lower repetitions and intermittent restriction to sustain performance while limiting excessive fatigue. Collectively, these findings demonstrate that low-load BFR-RE consistently produces hypertrophic and strength adaptations across a range of athletic populations, with protocol variability primarily influencing the magnitude rather than the direction of response.

### 3.1. Manipulation of Acute Program Variables

#### 3.1.1. Volume (Repetitions and Sets)

A common set and repetition scheme used in the literature involves a cumulative total of 75 repetitions across four sets of exercises, with 30 repetitions in the first set and 15 repetitions in each subsequent set (75-rep protocol) [12,19,20]. Another common scheme is 3–5 sets to concentric failure [1,21]; however, in applied settings, repetitions to absolute failure may not be necessary. In contrast, in KAATSU protocols, the first set typically targets 25 to 30 repetitions, followed by descending repetition counts according to accumulated fatigue, and the fourth set is optional [22]. Overall, the goal is to perform a repetition maximum to a point of local muscle failure while maintaining proficient technique. The associated lactate accumulation leads to transduction of growth factors that assist with the physiological changes observed with BFR-RE (as detailed in Part 1 of this article series) [23,24]. Subsequent sets should result in a decrease in repetitions, and this should be monitored and regulated in conjunction with control parameters and termination criteria. Furthermore, in applied strength and conditioning practice, athletes typically complete multiple exercises within a single session, in contrast to the majority of BFR-RE laboratory-based studies that examine only one or two exercises in isolation (see Table 1).

Consequently, a practical modification in applied settings would be to select a load that allows the athlete to complete 15 repetitions per set with good technique, as an alternative to rigid adherence to the canonical 75–rep protocol [13,14,25]. This approach provides sufficient metabolic stress to promote anabolic signaling and hypertrophic adaptations while potentially reducing the monotony and extended session duration by applying a fixed 75–repetition protocol to every exercise within a training session. Such considerations may influence athlete adherence and training consistency, though systematic investigation of psychological responses (e.g., enjoyment, motivation) to different BFR-RE protocols in athletic populations is limited and warrants future research. Direct comparisons between fixed-repetition (30-15-15-15) and failure-based protocols in athletic populations are currently lacking; therefore, practitioners should monitor individual responses when deviating from established protocols.

#### 3.1.2. Inter-Set Rest

Inter-set rest periods utilized during low-load BFR-RE are typically short and involve maintaining a continuous cuff pressure restriction throughout the training sets [26]. Physiological responses are potentiated through the accumulation of metabolites and intracellular swelling under sustained vascular restriction [27,28]. Thus, rest periods of both 30 s [29,30] and 30–60 s [31,32] are common within the BFR-RE literature. Although both continuous and intermittent pressure during rest periods have demonstrated positive effects, continuous pressure is recommended in low-load BFR-RE (as tolerated) to maximize the metabolic and cell-swelling stress for adaptation [26].

#### 3.1.3. Frequency

Traditional (non-BFR) resistance training is often prescribed two to three sessions per week; however, low-load BFR-RE can be performed more frequently without impairing performance. High-frequency training (e.g., 4–5 sessions per week) has been shown to accelerate hypertrophy and strength development [1,33,34]. Similarly, short-term (2 weeks) twice-daily BFR-RE has produced significant increases in muscle strength and hypertrophy [2,13,35], which may be advantageous during periods of intensive conditioning or rehabilitation [36]. Table 2 presents clear guidelines for low-load BFR-RE prescription. These reflect a consistent evidence-based approach and practical reference for the manipulation of the acute program variables within athletic programs. The following guidelines represent a synthesis of the available evidence alongside established BFR practice and should be interpreted as applied recommendations rather than prescriptive protocols.

## 4. Moderate to High Load BFR Resistance Exercise (> 50% 1RM)

While the majority of BFR-RE research has focused on low-load training protocols (20–40% 1RM), an emerging body of literature has investigated the acute and longitudinal effects of applying BFR during moderate to high-load resistance exercise (>50% 1RM) (Table 3). Most acute BFR-RE studies utilizing moderate to high loads have focused on barbell velocity as the primary outcome, reflecting its strong relationship with power output and neuromuscular performance. The findings reveal a complex, load-dependent relationship between BFR applications, cuff width, and cuff inflation mode (i.e., continuous vs. intermittent).

### 4.1. Barbell Velocity

Several investigations have demonstrated that the addition of BFR can maintain or even enhance barbell velocity under specific conditions. Wilk et al. [5] examined squat performance across multiple loads (40–90% 1RM) using both intermittent and continuous BFR-RE protocols. Although there were no statistical differences between BFR-RE protocols, small to moderate effects were noted in favor of wearing BFR cuffs. Notably, at 90% 1RM, intermittent BFR produced a moderate velocity improvement (ES: 0.67) compared to no-BFR conditions, suggesting that properly timed cuff restriction-reperfusion cycles may improve barbell velocity at maximal loads

### 4.2. Intermittent and Continuous Cuff Inflation

In a similar study design investigating upper body responses, bench press velocities were compared using an intermittent (I-BFR) and continuous (C-BFR) cuff inflation. There was a significant effect for peak barbell velocity for both the C-BFR and I-BFR conditions when compared to the control (no-BFR) [4]. Despite barbell velocities at 50%, 60%, 80%, and 90% 1RM for both BFR conditions being faster than the no-BFR condition, only the 50% 1RM load reached significance. The practical application aligns with the broader velocity-based training literature, demonstrating that training with maximal intended velocity produces superior adaptations in strength, power, and speed compared to slower movements [40]. For practitioners, providing velocity-based feedback may help maintain movement quality and neural drive during BFR-RE; however, this application has not been empirically tested.

### 4.3. Fatigue and Recovery Considerations

A concern when performing high-load BFR-RE using maximal repetitions is the potential for excessive fatigue accumulation. Studies examining muscle fatigue during high-intensity BFR-RE have reported increased ratings of perceived exertion and greater metabolite accumulation (i.e., lactate, hydrogen ions) [6,7]. Despite these findings, there were no post-exercise performance decrements as a result of the fatiguing stimulus when compared to the control (no-BFR) condition. Therefore, intermittent BFR cuff inflation is recommended to mitigate fatigue-related impairments compared to continuous cuff restriction, likely due to improved metabolite clearance and oxygen resupply during rest periods [6]. This has important practical implications for programming BFR-RE with high loads, suggesting that intermittent protocols may offer a better balance between metabolic stress and performance preservation. Importantly, differences in reported fatigue and performance outcomes across studies are likely explained by variations in protocol design. Higher-volume or AMRAP-based protocols tend to induce greater metabolic stress, perceptual fatigue, and discomfort, whereas lower-volume, strength-oriented protocols appear to maintain performance without excessive fatigue accumulation. Therefore, these findings should not be viewed as contradictory, but rather as reflective of the interaction between BFR application and program design.

### 4.4. Program Variable Guidelines

Table 4 presents guidelines for moderate-to-high load BFR-RE prescription. These recommendations are consistent with manipulation of established acute program variables and strength-training principles, while incorporating BFR-specific considerations. These studies suggest that moderate-to-high-load BFR-RE does not impair performance and may enhance velocity and power under specific loading and cuff-application conditions, although responses appear load- and protocol-dependent. The following guidelines represent a synthesis of the available evidence alongside established BFR practice and should be interpreted as applied recommendations rather than prescriptive protocols.

## 5. Non-Local Adaptations to BFR-RE

The preceding sections have focused on direct adaptations within the limb exposed to BFR. However, BFR can influence musculature beyond the cuff site, offering expanded applicability in situations where unilateral injury, immobilization, or loading restrictions impose constraints. Two key phenomena, contralateral/cross-transfer effects and proximal/distal adaptations, demonstrate that BFR can facilitate improvements in muscle size and strength adaptation in regions not directly placed under restriction.

### 5.1. Contralateral and Cross-Transfer Effects

The application of BFR to unilateral training has been investigated through two distinct paradigms: direct contralateral effects and cross-transfer effects. The concept of contralateral strength training describes the phenomenon where training one limb improves strength in the contralateral homologous muscle group [41]. This has been well studied with respect to conventional (non-BFR) resistance training, with meta-analytic data indicating that performing unilateral resistance training at intensities ≥ 50% 1RM for at least 2 weeks results in moderate contralateral strength gains (~7.8%, ES~0.5–0.6) in healthy young adults [41]. This cross-education effect appears independent of contraction mode (i.e., isometric vs. dynamic) and applies to both upper- and lower-limb musculature [41].

Within BFR research, two related phenomena have been described. Direct contralateral effects refer to adaptations in the contralateral homologous limb when BFR is applied during unilateral training. In contrast, cross-transfer effects describe adaptations that occur when BFR is applied to one body region (e.g., lower body) and enhances training responses in a different body region (e.g., upper body) that is also being trained [33].

#### 5.1.1. Evidence for Contralateral Effects

Low-load BFR-RE training has demonstrated contralateral effects for strength-related outcomes, in both the upper [42,43] and lower [43,44] body when compared to low-load training alone. For the upper body, recreational-level athletes completed 6 weeks of bilateral shoulder and elbow exercises with BFR applied to one limb, while a control group performed the same program without BFR [42]. Despite significant improvements in the BFR restricted limb, the only significant contralateral effect was observed in grip strength, with the non-BFR limb demonstrating a 6 ± 2% increase compared to a 3 ± 3% decrease in controls (*p* < 0.01) [42]. No significant contralateral changes were observed in shoulder strength measures despite small numerical increases.

Using a similar unilateral training design, 6 weeks of lower-body resistance exercise resulted in significant improvements in lower-body strength and circumference in the BFR limb. Contralateral effects were observed for thigh circumference (2.3 ± 1.6% vs. 0.8 ± 2.0% in controls, *p* = 0.01), knee extension peak torque (8 ± 9% vs. 3 ± 9%, *p* = 0.04), and single-leg heel raises (16 ± 18% vs. 4 ± 18%, *p* = 0.02) [44]. However, substantial inter-individual variability was reported, with standard deviations often approaching or exceeding mean changes, indicating inconsistent responses across participants.

#### 5.1.2. Evidence for Cross-Transfer Effects

Cross-transfer effects represent a distinct phenomenon where BFR applied to one body region during exercise enhances training adaptations in a different body region that is also being trained [33,43,45]. To illustrate this, Madarame et al. used a design in which participants performed unilateral arm curls (3 × 10 repetitions at 50% 1RM) while the opposite arm remained untrained, followed by lower-body resistance exercise (knee extension and flexion; 30/15/15 repetitions at 30% 1RM) with or without BFR applied to the legs [33].

After 10 weeks of training, significant increases in muscle size and isometric strength were observed only when arm training was combined with BFR leg exercise, whereas the untrained contralateral arm showed no significant changes. In the non-BFR condition, arm training improved 1RM strength, but did not produce significant hypertrophy or isometric strength gain. The untrained contralateral arm showed no significant changes in either group, indicating that the effect was not a direct contralateral transfer but rather a cross-transfer enhancement of the trained limb’s response [33].

This cross-transfer effect has been hypothesized to arise from a combination of systemic hormonal and metabolic responses induced by BFR. Madarame et al. reported significantly elevated post-exercise noradrenaline levels in the BFR group compared to controls (*p* < 0.05), while growth hormone showed a trend toward elevation that did not reach statistical significance (*p* = 0.12) [33]. These findings suggest that systemic factors may augment training responses in muscles that are being actively trained, even when BFR is applied to a different body region. However, the lack of adaptation in the untrained contralateral arm highlights that local mechanical loading remains important for meaningful strength and hypertrophy adaptations, even in the presence of elevated systemic anabolic factors [33]. The relative contribution of neural versus hormonal mechanisms remains unclear, and the inconsistent findings across muscle groups suggest that these effects may be influenced by factors such as muscle size, training status, and proximity to the site of BFR application.

#### 5.1.3. Practical Implications

Despite the limited and inconsistent evidence for direct contralateral effects, the application of BFR to the contralateral limb or to a different body region may have practical utility in specific clinical contexts. In operative and non-operative orthopedic conditions where direct training of an injured limb is contraindicated or not tolerated, contralateral or cross-transfer BFR training may provide a modest protective effect against strength loss and muscle atrophy. The cross-transfer approach, where BFR is applied to an uninjured body region during exercise while also performing low-intensity exercise of the injured limb (when appropriate), may be particularly promising given the evidence that systemic factors can enhance training responses in muscles receiving direct mechanical stimulus.

Practical implementation of contralateral or cross-transfer BFR training requires careful consideration of injury status, loading tolerance, and the rehabilitation stage. Clinicians should recognize that these approaches are unlikely to fully prevent detraining and should be viewed as adjunctive strategies rather than replacements for direct training when feasible. Table 5 provides scenario-specific guidelines for applying BFR to maximize potential cross-education and cross-transfer effects while respecting injury-related constraints. The following guidelines are based on a synthesis of the available evidence and the applied experience of the authors and should be interpreted as expert-informed recommendations rather than prescriptive protocols.

### 5.2. Proximal and Distal Effects

It has been originally speculated that the direct muscular effect from the BFR stimulus is distal to the location of the pressure cuff and that the muscles that are proximal or not placed under direct restriction may not see any benefit from the application of BFR [46]. However, BFR-RE for both upper and lower body has reported increases in muscle size and recruitment located both proximal and distal to the placement of the cuff, although methodological heterogeneity across studies (e.g., cuff width, inflation pressure, training intervention) affects direct comparison and may contribute to variability in reported outcomes.

In upper body resistance exercise training, several studies have reported significant increases in triceps brachii (4.9–8%) and pectoralis major (8.3–16%) muscle hypertrophy following low-load (30% 1RM) BFR bench press performed under two distinct protocols: a short-term high-frequency protocol (twice daily, 6 days·week^−1^ for two weeks) and a moderate-frequency protocol (3 sessions·week^−1^ for six weeks) [12,47,48]. For example, the addition of BFR to low-load bench press (30% 1RM; 75-repetitions) using a short-term high-frequency protocol resulted in the pectoralis major and triceps brachii muscle thickness increased 16% and 8% (*p* < 0.01) respectively, but not in the control condition (2% and −1% respectively) [12]. Similarly, 3 weeks of low-load bench press using a moderate-frequency protocol reported an increase in pectoralis major and triceps brachii muscle thickness (8.3% and 4.9%, *p* < 0.01) [48].

In lower-body BFR-RE squat training (20% 1RM, twice daily, 6 days·week^−1^ for two weeks), the gluteus maximus (which acts synergistically during the squat exercise) significantly increased in muscle size (9.1%) concomitantly with increases in the quadriceps (7.7%) and biceps femoris (10.1%) muscle groups [14]. Performing 14 weeks of low load BFR-RE calf raises (seated and standing; 20–35% 1RM; 30-15-15-15; 3 days·week^−1^) with the BFR cuffs placed on the thigh, resulted in significant increases in gastrocnemius medialis muscle cross-sectional area (9.1%), plantar flexor strength (9.8%) as well as significant increases in Achilles tendon stiffness (36.1%) and tendon cross-sectional area (7.8%) [49]. The observed changes were comparable with a high load training group (70–85% 1RM; 3 × 6–12; 7.7%, 13.5%, 40.7%, 4.6% respectively). Aside from the distal effect on muscle hypertrophy and strength, the additional findings from this study provide evidence that low-load BFR-RE may increase tendon mechanical and morphological properties to a similar extent as conventional high-load RE. This may be of particular importance for individuals who may not tolerate high-load training but still require improvements in myotendinous function. Based on these findings, BFR-RE may represent a potential alternative for treating tendinopathies in individuals unable to tolerate high-load isometric and eccentric resistance exercise [50,51,52]; however, its application in tendinopathy management is based on mechanistic rationale rather than direct evidence.

These findings illustrate that BFR-RE can increase muscle size and strength of various muscle groups, both proximal and distal to the cuff placement. These changes are not strictly limited to the limbs and the muscle groups that are directly distal to the cuff placement, but rather are reflective of the muscles that are activated through exercise. Therefore, exercise selection should consider the muscle groups that need to be targeted. Additionally, these studies have also highlighted that these muscle groups may require additional repetitions or progressive loadings to elicit significant changes that are comparable to higher loading parameters.

The mechanisms underlying these divergent patterns of proximal versus distal adaptations to BFR are not well understood. Proposed explanations include systemic hormonal responses, local metabolic stress, and mechanical loading effects, but these mechanisms have not been directly tested in the context of these studies. It has been proposed that in multi-joint tasks, distal fatigue may increase the force demand on more proximal muscles, potentially increasing mechanical tension and motor unit recruitment across the kinetic chain; alternatively, local paracrine signaling from the BFR application may contribute to adaptations in nearby tissues [12,47,48]. However, these ideas remain speculative and require direct measurement during BFR resistance exercise.

## 6. Practical Recommendations

For coaches and practitioners implementing BFR-RE, it is recommended to begin with well-established low-load protocols before progressing to more advanced applications. Wherever possible, cuff pressures should be individualized using objective measurement, and BFR-RE should be integrated strategically within periodized training programs to complement, rather than replace, traditional resistance training. Rigorous safety screening and contraindication assessment should be applied, as outlined in Part 1, alongside ongoing monitoring of individual responses to allow protocols to be adjusted as required, with athletes appropriately educated regarding the expected sensations and perceptual demands associated with BFR-RE. Importantly, BFR-RE should be viewed as a complementary training tool that expands programming options, providing coaches and practitioners with flexibility to maintain training-induced adaptations during periods when conventional high-load training is limited, contraindicated, or strategically reduced.

## 7. Conclusions

BFR-RE represents a validated training modality for coaches and practitioners working with athletic populations across the full loading spectrum. Evidence from several controlled studies indicates that low-load BFR-RE (20–50% 1RM) can elicit hypertrophy and strength adaptations approaching those observed with traditional high-load methods while reducing overall mechanical stress. These characteristics make low-load BFR-RE particularly suited to rehabilitation, in-season maintenance, congested competition periods, and situations in which joint loading must be constrained. Moreover, low-load BFR-RE offers additional utility when integrated as supplementary hypertrophic volume following traditional strength sessions or during deloads and high-frequency training blocks. For moderate-to-high loads (>50% 1RM), evidence supports BFR use with strength and power objectives. When strategically implemented, the addition of BFR can preserve or improve barbell velocity and facilitate neuromuscular qualities relevant to velocity-based training. Although the evidence base is emerging, such findings indicate that BFR-RE can complement, rather than replace, conventional strength programming in advanced athletes.

The inclusion of contralateral cross-education effects and proximal–distal adaptations expands the physiological outcomes of BFR-RE in circumstances involving unilateral injury, immobilization, or restricted limb loading. Contralateral training effects demonstrate ipsilateral strength preservation or enhancement during unilateral BFR-RE compared to controls. While these effects are smaller than direct training, they offer practical utility during injury rehabilitation when direct training of the affected limb is contraindicated. Likewise, proximal–distal adaptations confirm that hypertrophic and tendon-related responses are driven by recruitment demands rather than distal cuff placement alone, permitting targeted intervention in musculature that may not directly lie beneath the cuff.

Despite these advantages, several implementation considerations remain. Appropriate screening, accurate determination of individualized arterial occlusion pressure, informed cuff selection, progressive familiarization, and active monitoring of perceptual responses are required to ensure safety and efficacy. Furthermore, long-term effects and performance transfer to sport-specific outcomes remain understudied. BFR-RE should be positioned as a complement to established loading strategies, acknowledging that maximal-strength development in athletic populations still requires exposure to traditional high-load training where it is tolerated. Collectively, BFR-RE across the loading spectrum provides coaches and practitioners with an adaptable training tool that promotes morphological and neuromuscular adaptations under conditions where conventional loading is not feasible or must be modified. The strategic use of BFR-RE, guided by individual constraints, performance objectives, and phase-specific demands, will enable practitioners to apply this method confidently within athletic populations.

## Figures and Tables

**Table 1:** Low-load BFR Resistance Exercise Training Studies in Athletic Populations (≤50% 1RM).

Study	Population	Cuff Pressure Width	Protocol	Key Findings	Applied Insights
Takarada et al. [1] *Randomized 3 groups*	Elite male rugby players male: BFR *n* = 6; CON *n* = 6; Nil *n* = 6	200 mmHg 3.3 cm cuff	Bilateral knee extension: 50% 1RM; 8 wks; 2 × wk; 4 sets to failure, 30 s rest	🠝 Isokinetic strength 14.3% ^1^ 🠝 MVC30 9.3% ^1^*^##^ 🠝 CSA 12.3% ^1^	BFR effective adjunct for hypertrophy and strength; useful in-season.
Abe et al. [2] *Randomized 2 groups*	Collegiate track and field athletes (male): BFR *n* = 9; CON *n* = 6	160–240 mmHg 5 cm cuff	Squat and leg curl: 20% 1RM; 8 days; 2 × day; 3 × 15 reps; 30 s rest	🠝 1RM leg press 9.6% ^1^ (CON 4.8%) 🠝 MTH: 4.5–5.9% ^1^ 🠝 10m/30m sprint times^1^	High-frequency BFR protocols effective for improvements in functional strength and performance. Effective in-season option.
Manimmanakorn et al. [3] *Randomized 3 groups*	Female netball athletes: BFR *n* = 10; HT *n* = 10; CON *n* = 10	160–230 mmHg 5 cm cuff	Bilateral knee extension: 50% 1RM; 8 wks; 2 × wk; 4 sets to failure, 30 s rest	🠝 MVC3 13.3% ^1^*^#^ 🠝 muscular endurance ^1^ 🠝 Reps20 87.7% ^1^*^##^ 🠝 CSA 6.6% ^1^*^#^	BFR improves strength/endurance with low loads resulting in similar gains to HT; superior to CON.
Yamanaka et al. [15] *Randomized 2 groups*	Collegiate Division I male football players: BFR *n* = 16; CON *n* = 16	Elastic wraps 5 cm cuff	Bench press and squat: 20% 1RM 4 wks; 3 × wk; 30-20-20-20 reps, 45 s rest; supplemental post-HL-TRT	🠝 1RM Bench 7.0% * (CON 3.2%) 🠝 1RM Squat 8.0% * (CON 4.9%) 🠝 Chest girth 3.5% * (CON 0.9%)	Low load BFR used post heavy training provides additional hypertrophy/strength stimulus in well-trained athletes.
Luebbers et al. [16] *Randomized 3 groups*	Adolescent Weightlifters Age: 14–17 (22 boys; 3 girls): BFR *n* = 8; CON *n* = 8; HL *n* = 9	Elastic wraps 7.6 cm cuff	Squat exercise: 6 wks, 2 × wk, 45 s inter-set rest; BFR and CON: 20%1RM, 30-15-15-15 reps; 30 s rest; HL: 3 × 10 (65–75% 1RM); 3 × 3 (80–90% 1RM); 3–5 min rest	🠝 1RM Squat +14.2 kg ^1^ (HL +6.6kg)	Low load BFR is appropriate for youth athletes with similar strength gains with reduced loading demands.
Luebbers et al. [17] *Randomized 3 groups*	Collegiate male football players: BFR (HL+S) *n* = 17; CON (HL+S) *n* = 14; HL (only) *n* = 15	Elastic wraps 7.6 cm cuff UB and LB	Bench press and squat: 20% 1RM 7 wks; 4 × wk; 4 sets 30-20-20-20; 45 s rest; supplemental after HL program	🠝 1RM Squat: BFR(HL+S) 24.6 kg *^1^ 🠝 1RM Bench Press: BFR(HL+S) 24.4 kg ^1^	Addition of a BFR supplementary program to a traditional HL resistance program is effective to improve 1RM strength.
Bjørnsen et al. [18] *Randomized 2 groups*	National-level powerlifters: BFR *n* = 9; CON *n* = 8	Elastic wraps ~120 mmHg 13 cm cuff	LL-BFR vs. CON (HL-TRT) Front Squat: 24–31% 1RM 4 sets: AMRAP-15–12-AMRAP Wk 1 and 3, 5 × wk; CON: 60–85% 1RM; 6–7 sets; 1–6 reps	🠝 Type I CSA: 12% 🠝 Myonuclei: 18% 🠝 VL CSA: 7.7%	BFR useful during deloads, taper phases, or joint-sensitive periods while maintaining hypertrophic stimuli.

Abbreviations: BFR, blood flow restriction; CON, control; cm, centimeters; 1RM, one repetition maximum; mmHg, millimeters of mercury; MTH, muscle thigh thickness; m, meters; s, seconds; CSA, cross sectional area; HT, hypoxic training; ext/flex, extension and flexion; MVC3, maximal voluntary contraction for 3 s; MVC30, maximal voluntary contraction for 30 s; Reps20, number of repetitions at 20% 1RM; HT, hypoxic training; HL+S, high load and supplemental program; Mod+S, modified and supplemental program; UB, upper body; LB, lower body; HL, high load; TRT, traditional resistance training; HL-TRT, high load traditional resistance training; AMRAP, as many repetitions as possible; reps, repetitions; VL, vastus lateralis; RE, resistance exercise; 🠝, increase; 🠟, decrease; *, *p* < 0.05 compared to control; ^1^, *p* < 0.05 compared to pre-training values; ^#^, small effect size compared to control; ^##^, moderate effect size compared to control.

**Table 2:** Guidelines for Low-Load BFR-RE: Acute Program Variables.

Variable	Guidelines	Applied Insights
Frequency	2–3 × week (>3 weeks) or 1–2 × day, 4–5 × /week (≤2 weeks)	High frequency BFR-RE will predominantly focus on loads of 20–40% 1RM.
Load	20–50% 1RM	
Exercise type	Upper and lower body Unilateral and bilateral	Small and large muscle groups.
Sets	2–4 sets	Guided by termination criteria and number of exercises.
Repetitions	1–2 exercises: 75 rep protocol (30-15-15-15), or S1 = 20–30 reps; S2–4 = 10–15 reps (S4 optional) ≥3 exercises: 3 sets × 15 reps	Descending repetitions across sets. Execute rep and set scheme until concentric failure or when planned rep scheme is completed. Practical suggestion: Select a load that yields ~15 reps in S1; if >18 reps are achieved, consider increasing load in the subsequent session.
Cuff pressure	50–80% AOP	Gradient pressure according to cuff width, material and athlete tolerance.
Rest between sets	30–60 s	
Pressure restriction	Continuous or intermittent	
Restriction time	Upper body: 15 min Lower body: 20 min	Time in one continuous bout. May be reinflated following reperfusion if additional exposure is required; cumulative restriction time should be considered relative to safety and individual tolerance.

Abbreviations: RM, repetition maximum; %, percentage; AOP, arterial occlusion pressure; BFR, blood flow restriction; RE, resistance exercise; rep, repetition; S, set; min, minutes.

**Table 3:** Moderate- to High-load BFR Resistance Exercise Training Studies in Athletic Populations (>50% 1RM).

Study	Population	Cuff Pressure Width	Protocol	Key BFR Findings	Applied Insights
Wilk et al. [5] *Randomized crossover 2 groups*	Resistance-trained males (*n* = 11); Back squat 1RM ≥ 150% BW	~80% AOP 10 cm cuff	Back squat: 40–90% 1RM 6 × 2 reps; 3 min rest I-BFR, C-BFR, CON	NS difference between groups. 70%1RM: I-BFR ^#^; 80%1RM: C-BFR ^##^ 90%1RM: I-BFR ^##^ 80–90%1RM: I-BFR > C-BFR ^^^	BFR does not impair performance across a wide loading range. I-BFR may be preferential over C-BFR.
Wilk et al. [4] *Randomized crossover 3 groups*	Resistance-trained males (*n* = 11); Bench press 1RM ≥ 120% BW	70% AOP 4 cm cuff	Bench press: 20–90% 1RM 8 × 2 reps; 3 min rest I-BFR, C-BFR, CON	🠝 PV 50% 1RM: I-BFR * C-BFR *	BEF effects persist at higher loads (>80% 1RM); consider individualized load-specific BFR.
Wilk et al. [37] *Randomized crossover 3 groups*	Resistance-trained males (*n* = 14); Bench press 1RM ≥ 120% BW BFR-N; BFR-W; CON	90% AOP 4 cm cuff (BFR-N) 10 cm cuff (BFR-W)	Bench Press: 70% 1RM 1 × 3 reps	BFR-W vs. CON: 🠝PV 22% *^###^ 🠝 MV 21% *^###^ BFR-N vs. CON: 🠝PV 2% ^#^ 🠝MV 3% ^#^ BFR-W vs. BFR-N: 🠝PV 18% *^###^ 🠝 MV 13% *^###^	BFR cuff width meaningfully influences bar velocity, due to potential mechanical energy storage.
Hornikel et al. [6] *Randomized crossover 2 groups*	Advanced resistance-trained participants (*n* = 13; 4 female; 9 male)	80% AOP 11.5 cm cuff	Squats: 75% 1RM 4 × AMRAP; 3 min rest; C-BFR between sets 1–2 and 3–4.	🠟 total reps: 25.8 * (CON 42.2) 🠝 pain across all sets * 🠝 IL-6 39.7% * 🠟 [La−]b at 2-min post-exercise 🠟 CMJ 🠜🠞 vs. CON	BFR with AMRAP induces early fatigue and higher discomfort; best for advanced athletes seeking strong hypertrophic stimulus. Mechanism justification for hypertrophy and strength gains.
Neto et al. [7] *Randomized crossover 2 groups*	Trained male Jiu-Jitsu fighters (*n* = 12)	60% AOP Cuff width not reported	Squat: 80% 1RM 1 × AMRAP	🠟EMG (VL, VM) ^1^ 🠟MVIC both conditions	BFR induces similar acute fatigue to non-BFR; does not impair performance relative to heavy training.
Wang et al. [38] *Randomized 3 groups*	Trained male volleyball players (*n* = 18); HL-BFR *n* = 6; LL-BFR *n* = 6; HL-TRT *n* = 6	180 mmHg 7 cm cuff	Half Squat: 8 wk; 3 × wk; 60 s rest; HL-BFR & HL-TRT: 70% 1RM, 4 × 8 reps; LL-BFR: 30%1RM, 30-15-15-15 reps	HL-BFR: 🠝1RM Half Squat 28.6% ^1^, 🠝Knee flexion ~17% ^1^ & ext ~16% ^1^ 🠝SJ ^1^ 🠝 3FT ^1^ Overall: HL-BFR > HL-TRT > LL-BFR ^2^ LL-BFR: 🠝1RM Half Squat 9.9% ^1^ Peak Torque—Knee flexion 5–9% ^1^	The addition of BFR to HL squats appears to be a superior training methodology for strength and jumping performance. LL-BFR remains effective when high loads are contraindicated.
Cook et al. [39] *Randomized 2 groups*	Semi-professional rugby male (*n* = 20)	180 mmHg 10.5 cm cuff I-BFR (thighs only)	Squat, bench press, pull-up: 70% 1RM; 5 × 5 reps; 90 s rest; 3 wk; 3 × wk	BFR: 🠝1RM Squat * 🠝1RM Bench Press * 🠝CMJ Peak Power * 🠝40m Sprint * 🠝40m RST *, 🠝salivary testosterone *	Systemic benefits including upper-body 1RM improvements. BFR applied to the lower limbs can modulate adaptation in the upper body.

Abbreviations: BFR, blood flow restriction; CON, control; BFR-N, narrow cuff width; BFR-W, wide cuff width; BW, body weight; I-BFR, intermittent blood flow restriction; C-BFR, continuous blood flow restriction; LL, low load; HL, high load; TRT, traditional resistance training; HL-TRT, high load traditional resistance training; RM, repetition maximum; CMJ, countermovement jump; VL, vastus lateralis; VM, vastus medialis; RST, repeat sprint test; ext, extension; SJ, squat jump; 3FT, 3 foot take-off; VJ, vertical jump; s, seconds; min, minutes; NS, non-significant; ^#^, small effect size compared to control; ^##^, moderate effect size compared to control; ^###^, large effect size compared to control; ^^^, moderate effect size; IL-6, interleukin-6; [La−]b, blood lactate; 🠝, increase; 🠟, decrease; 🠜🠞, no change; NS, no significant difference (*p* > 0.05); ^1^, *p* < 0.05 compared to pre-training values; ^2^, *p* < 0.05 HL-BFR; *, *p* < 0.05 compared to control.

**Table 4:** Guidelines for Moderate-to-High BFR-RE: Acute Program Variables.

Variable	Guidelines	Applied Insights
Frequency	1–3 sessions per week	Integrate within existing strength blocks; monitor fatigue when pairing with high neural-demand exercises.
Load	50–90%+ 1RM	Traditional strength and power loading principles still apply. Warm-up sets (<50% 1RM) may be performed under continuous inflation; then transition to intermittent pressure for heavier sets.
Exercise type	Multi-joint and single-joint lifts	Minimal to no change in exercises. Use VBT feedback or intent-based cueing to enhance movement velocity.
Sets	3–5 working sets	Matches traditional strength protocols.
Repetitions	2–8 reps per set	Align with strength goals. Practical suggestion: cluster set ^†^ formation may be considered (derived from general strength training principles; not tested in BFR contexts).
Cuff pressure	50–80% AOP	Gradient pressure according to cuff width, material and athlete tolerance.
Rest between sets	3–5 min	Ensure sufficient recovery to maintain lifting quality.
Pressure restriction	Intermittent for heavy sets; continuous optional for warm-up sets	Continuous pressure in warm-up may enhance priming effects (not directly tested in BFR contexts); intermittent pressure preserves bar velocity during heavy sets.
Restriction time	Upper body: 15 min Lower body: 20 min	Time in one continuous bout. Intermittent inflation mode during heavy sets will negate the concern for restriction time.

Abbreviations: RM, repetition maximum; %, percentage; AOP, arterial occlusion pressure; BFR, blood flow restriction; RE, resistance exercise; rep, repetition; S, set; min, minutes; VBT, velocity-based training; ^†^ cluster set, intra-set rest intervals (~30 s) to allow partial recovery and maintain movement velocity/quality.

**Table 5:** Practical Guidelines for Implementing Contralateral BFR Training.

Scenario	Guidelines	Applied Insights
Acute Phase (2–3 wk)	High-frequency BFR-RE following low-load guidelines (Table 2); 2–6 × wk if tolerated.	Goal is to attenuate muscle and strength loss.
Immobilized Limb	Train the uninjured limbs only; apply BFR according to LL-BFR guidelines.	Cross-education can attenuate muscle strength and size loss in immobilized limb.
Limb in a Cast	Exercise: Unilateral training of the uninjured limb; BFR on uninjured limb only. Passive BFR-based interventions may be considered once medically cleared.	If appropriate, bilateral exercises may still be performed with modified loading on the injured limb, and opposing limb training (upper-lower) should be maintained to maximize neuromuscular stimulus.
No Cast (restricted loading)	Begin bilateral BFR-RE once musculoskeletal milestones allow.	Introduce low-load BFR first; progress to moderate loads as tolerated.
3 wk+ Pending Rehabilitation and Musculoskeletal Milestones Have Been Met		
Injured limb	Continued rehabilitation progression; integrate progressive BFR-RE to restore strength.	Frequency: 1–2 ×/day according to rehabilitation requirements.
Uninjured limb	Integrate moderate-to-high load BFR-RE for the uninjured and opposing limb.	Frequency 2–4 ×/wk within standard strength periodization.

Abbreviations: BFR, blood flow restriction; RE, resistance exercise; wk, week; IPC, ischemic preconditioning.

## Data Availability

As this is a narrative review, no new data were generated. All data discussed are available in the cited original publications.

## Abbreviations

The following abbreviations are used in this manuscript:

AOP	arterial occlusion pressure
BFR	blood flow restriction
CON	control
C-BFR	continuous blood flow restriction
ES	effect size
HT	hypoxic training
HL	high load
TRT	traditional resistance training
HL+S	high load and supplemental program
I-BFR	intermittent blood flow restriction
LB	lower body
LL	low load
LL-BFR	low-load blood flow restriction
MTH	muscle thigh thickness
mmHg	millimeters of mercury
PV	peak (barbell) velocity
rep/reps	repetition/repetitions
RM	repetition maximum
RST	repeat sprint test
SJ	squat jump
UB	upper body
VBT	velocity-based training
wk	week
1RM	one-repetition maximum
[La−]b	blood lactate
